# Supplementary material for: Prospective Validation of Indocyanine Green Lymphangiography Staging of Breast Cancer-Related Lymphedema
Source: Cancers (Basel). 2021 Mar 26;13(7):1540. doi: 10.3390/cancers13071540 (PMC8063087; doi:10.3390/cancers13071540)
Supplement: Supplementary file 1 [file cancers-13-01540-s001.zip › cancers-1126771-suppl.pdf]

Supplementary Material

Doi: 10.5281/zenodo.4524636

Link: https://doi.org/10.5281/zenodo.4524636

Supplementary ICG videos: Video S1: Stage 0, Video S2: Stage 1 (MDA), Video S3: Stage 2 (MDA), Video S4: Stage 2 (ADB), Video S5: Stage 3 (MDA), Video S6: Stage 3 (ADB), Video S7: Stage 4 (severe backflow), Video S8: Stage 4 (some backflow), Video S9: Stage 4 (minimal proximal backflow), Video S10: Stage 5 No flow.
